# Osteoarthritis with depression: mapping publication status and exploring hotspots

**DOI:** 10.3389/fpsyg.2024.1457625

**Published:** 2024-10-24

**Authors:** Meng Zhang, Hao Li, Qingshan Li, Zhen Yang, Haobin Deng, Yingying Xu, Quanyi Guo

**Affiliations:** ^1^School of Business, Renmin University of China, Beijing, China; ^2^School of Medicine, Nankai University, Tianjin, China; ^3^Institute of Orthopedics, The First Medical Center, Chinese PLA General Hospital, Beijing Key Lab of Regenerative Medicine in Orthopedics, Key Laboratory of Musculoskeletal Trauma & War Injuries PLA, Beijing, China; ^4^Department of Spinal Surgery and Translational Medicine, The Fourth Medical Center, Chinese PLA General Hospital, Beijing, China; ^5^Arthritis Clinical and Research Center, Peking University People's Hospital, Beijing, China; ^6^Department of Oncology, Liuzhou People’s Hospital Affiliated to Guangxi Medical University, Liuzhou, China

**Keywords:** bibliometric analysis, depression, osteoarthritis, evolving trends, CiteSpace

## Abstract

Depression is a common psychological complication in osteoarthritis (OA) patients, and its incidence gets more and more attention year by year worldwide. This study investigates the association between OA and depression through a bibliometric analysis of published studies. It aims to identify leading authors, institutions, and countries to highlight research hotspots and suggest potential future directions. We collected publications on OA and depression from 1994 to 2024 using the Web of Science Core Collection (WOSCC) database. Bibliographic information, including authorship, country of origin, citation frequency, and visualizations, was generated using VOSviewer, R software, and CiteSpace. A total of 2,342 articles were identified. The United States led in publications with 906 articles, Boston University was the most prolific institution with 56 publications, BMC Musculoskeletal Disorders was the top journal with 71 publications, and Stefania Maggi was the most productive author with 19 publications. The primary research hotspots identified were: “The relationship between depression and OA,” “Disability and prevalence,” and “Characteristics of older people suffering depression after OA.” Predicted future research frontiers include: “Treating depression in OA patients with multimorbidity,” “Psychometric properties of instruments for assessing depression and anxiety in OA patients,” “Depression or anxiety in patients with surgical intervention,” and “Other mental diseases in OA patients.” This bibliometric analysis underscores the importance of understanding the link between OA and depressive disorders, potentially guiding new research directions.

## 1 Introduction

Osteoarthritis (OA) is a prevalent condition that significantly contributes to disability, especially among older adults. It is the leading cause of disability in this age group, with a high prevalence of knee pain and radiographic OA in the general population (Peat et al., 2001). Global estimates from the Global Burden of Disease studies suggest that the true burden of OA may be underestimated due to methodological issue (Cross et al., 2014). Additionally, research in Colombia and Ecuador has estimated the burden of chronic pain, with OA being a significant contributor to disability-adjusted life years (DALYs) in these populations (Lasalvia et al., 2022; Lasalvia et al., 2023). Economically, OA represents a substantial burden, with total costs for arthritis, including OA, potentially exceeding 2% of the gross domestic product in some countries. These costs encompass direct medical expenses and indirect costs such as lost productivity and early retirement due to disability (Felson et al., 2000). Employees with OA are more likely to take sick leave and require job modifications due to pain and reduced physical function, leading to significant work limitations and absenteeism (Gelber, 2014). The socioeconomic impact of OA includes higher out-of-pocket health-related expenditures and costs due to lost productivity. OA patients often have at least one other chronic condition, which exacerbates both the economic and functional burden (Leifer et al., 2022). These findings underscore the urgent need for improved approaches to manage and mitigate the impact of OA worldwide.

However, compared to rheumatoid arthritis (RA), the relationship between OA and depression has been largely overlooked in previous studies. This oversight is significant as it neglects the comprehensive impact of OA on mental health and overall quality of life. Notably, OA patients were reported to have higher rates of perceived stress, depressed mood, and suicidal ideation compared to individuals without OA (Jung et al., 2018). After a long period of neglect, the number of studies documenting the prevalence of depression in OA has increased in the past decade. According to the National Health Interview Survey (2015–2017), the age-standardized prevalence of depressive symptoms in adults with arthritis was 12.1%, compared to 4.7% in adults without arthritis (Guglielmo et al., 2018). Specifically, the prevalence of depressive symptoms in OA was found to be 19.9% (Stubbs et al., 2016). Additionally, the prevalence of depression among patients treated with total shoulder arthroplasty increased from 5.1% in 2002 to 15.4% in 2012 (Mollon et al., 2016), representing an almost three-fold increase. A similar trend has been observed among patients with total knee arthroplasty (TKA) (Mollon et al., 2016). Given the large population of OA patients, even a small percentage experiencing depressive symptoms translates into a significant number of individuals. This underscores the need for comprehensive care that addresses both the physical and mental health aspects of OA (Wang and Ni, 2022).

*Depression* is defined as a psychiatric disorder that affects mood, behavior, and overall health (Davidson et al., 2002). It causes prolonged feelings of sadness, emptiness, or hopelessness, and a loss of interest in activities that were once enjoyed. There is growing evidence that depression negatively impacts prognosis, healthcare expenditures, and quality of life (Tsuji et al., 2019). Specifically, individuals with both OA and depression incur 38.8% higher direct medical costs compared to those with only OA (Agarwal and Sambamoorthi, 2015). Additionally, depression is a risk factor for adverse outcomes in OA patients. While some studies have found that preoperative depression is linked to poorer postoperative outcomes, such as increased postoperative pain (Pan et al., 2019; Lewis et al., 2015; Duivenvoorden et al., 2013; Singh and Lewallen, 2010), poor clinical and functional improvement (Hanusch et al., 2014; Kohring et al., 2018; Singh and Lewallen, 2014), higher infection rates (Browne et al., 2014; Ali et al., 2017; Yang et al., 2021), and lower satisfaction (Bierke et al., 2020). Therefore, we should focus on identifying patient characteristics associated with persistent depression and determining high-risk groups for ongoing depressive symptoms.

Bibliometrics, first established as a formal discipline by Pritchard (1969), has gained increasing attention with the rise of computers and the internet. The field of bibliometrics has seen a rise in studies analyzing various journals across different disciplines, focusing on the overall growth structure, publication quality, and citation landscape (Shukla et al., 2019). Bibliometrics employs quantitative methods, using mathematical and statistical techniques to analyze scientific publications. It offers a clear presentation of contributions, research hotspots, and future trends in a specific field (Gu et al., 2017). Despite the widespread interest in the relationship between OA and depressive disorders, few bibliometric studies on this topic have been published. CiteSpace, VOSviewer, and R bibliometrix are popular bibliometric visualization tools used for data analysis and visualization in both medical and psychological domains. CiteSpace can conceptualize knowledge domains by generating and visualizing co-occurrence network maps of contributors and keywords, as well as co-citation networks of cited authors, based on bibliographical records collected from the WOSCC (Gu et al., 2017). VOSviewer offers similar functions, presenting co-occurring contributions but in a more simplified map (van Eck and Waltman, 2010). In sum, these tools help researchers identify trends and gaps in the literature, providing valuable insights into the relationship between OA and depression. We also conducted an analysis using the bibliometrix R-Tool (Aria and Cuccurullo, 2017), a recent R-package that enhances bibliometric analysis by offering specialized tools for quantitative research in both bibliometrics and scientometrics. In this regard, R is a highly powerful and flexible statistical software environment, widely recognized for its open-source accessibility. It serves as a comprehensive suite of applications for data manipulation, computation, and graphical visualization (Rodríguez-Soler et al., 2020).

Conducting bibliometric research on this highly debated topic is significant as it provides visualized summaries of past publications and anticipates potential future directions, offering more value than a conventional review. A well-organized bibliometric study can save researchers time by highlighting key research frontiers. In this study, we utilized the WoSCC database to gather relevant scientific publications from the past 30 years (1994–2024). We applied R bibliometrix, CiteSpace, and VOSviewer for bibliometric and visual analyses to present global research trends and explore key hotspots and emerging directions, providing guidance for future studies.

## 2 Materials and methods

### 2.1 Data source and search strategy

**Figure 1:**
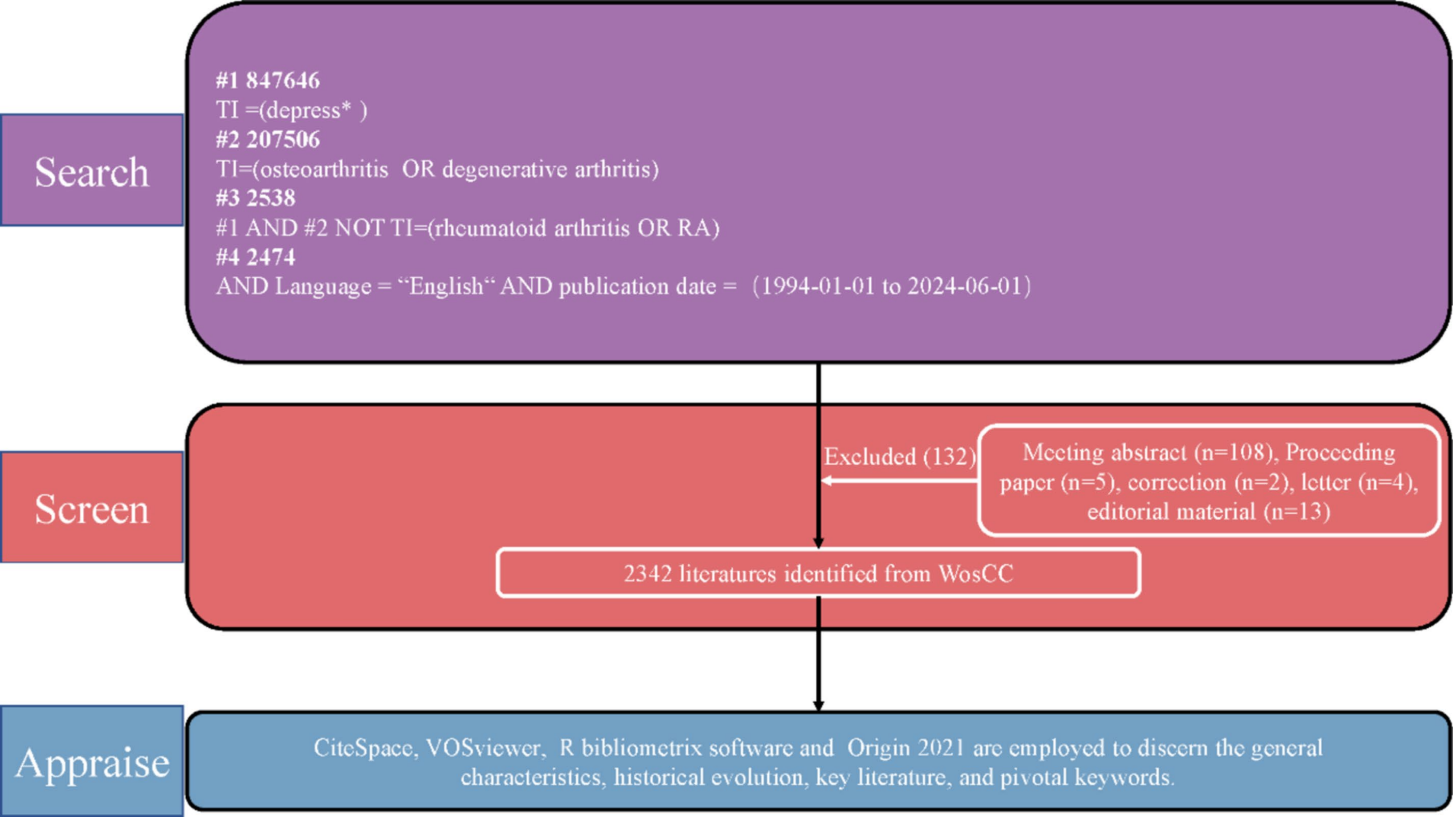
Flowchart illustrating the literature selection process.

Bibliometrics is a practical and effective method for quantitatively and qualitatively analyzing scientific publications. It provides essential information about the contributing authors, countries, and institutions, as well as emerging trends in the research field (Ellegaard and Wallin, 2015). Tools such as CiteSpace, VOSviewer, and R bibliometrix enhance bibliometric mapping, making it a valuable technique for analysis (Derviş, 2019; Yu et al., 2020; Su et al., 2019). The WoSCC database is considered the most suitable one for bibliometric analysis and is widely used for bibliometric analysis and visualization of scientific literature. We conducted a literature search on June 1, 2024, using the Web of Science Core Collection as our data source. The search terms were as follows: #1(depress*), #2(osteoarthritis OR degenerative arthritis), #3 = #1 AND #2 NOT TI = (rheumatoid arthritis OR RA), #4 = Language = “English” AND publication date = (1994-06-01 to 2024-06-01), as shown in Figure 1. The information for specific countries or regions in the WoSCC was refined by indexing them during the search process. All relevant data from the literature, including publication year, title, author names, nationalities, affiliations, abstracts, keywords, and journal names, were saved as download.txt files from the WoSCC database. Coauthors MZ and HL independently searched and extracted the data. Any discrepancies were resolved through expert consultation to reach a final consensus.

### 2.2 Bibliometric analysis and visualization

The primary function of WoSCC is to analyze the fundamental characteristics of relevant literature. This includes tracking the number of publications and their corresponding citations. The Relative Research Interest (RRI) represents the annual number of publications within a specific field relative to all publications in that field. A timeline of publication trends was created following the methodology of a previous study (Xing et al., 2018). The H-index, which quantifies the impact of a researcher’s work by indicating the number of papers (H) that have been cited at least H times, was used to measure scientific impact (Wu et al., 2022). Then, we utilized common bibliometric indicators in the scientific community to evaluate the obtained literature, such as total citations, average citations, and H-index (Hirsch, 2005). Journal impact factors (IF) were obtained from the Journal Citation Reports 2024 for our analysis.

This study utilized various software tools for the analysis and visualization of bibliometric data. CiteSpace was employed to analyze authorship patterns, active institutions, core journals, co-cited references, and time trends of significant keywords. In this analysis, node sizes represent the number of items, while different colors indicate different years. Specifically, we used CiteSpace (6.3.R2), developed by Professor Chen C, to examine country/region and institution collaboration, journal dual-map overlay, author collaboration and co-citation, co-cited literature, and keyword cluster detection, as well as to identify burst citations and keywords (Su et al., 2019). Additionally, VOSviewer (developed by Leiden University, Netherlands) was employed to form and visualize the bibliometric network of publications (van Eck and Waltman, 2010). In VOSviewer, distinct items are represented by nodes, where node size reflects the number of publications, node color indicates the publication year, and the thickness of connecting lines denotes the strength of collaboration or integration. In this study, this software was used to visualize the network, including country, institution, journal, author cooperation analysis, and keyword co-occurrence analysis. VOSviewer has powerful features for handling large maps, and it can display large bibliometric maps in an easily interpretable way (van Eck and Waltman, 2010). The R bibliometrix package was also employed for statistical computing and graphical analysis, offering strong extensibility for automating analyses and creating new functions. The cleansed data underwent bibliometric analysis using this package (Campra et al., 2022). Finally, all the coauthors independently cleaned and analyzed the data using Origin 2021 software. This software was primarily used to analyze and plot literature publication metrics, including the number of articles published per year, RRI, and H-index.

## 3 Results

### 3.1 Analysis of global publication trend

**Figure 2:**
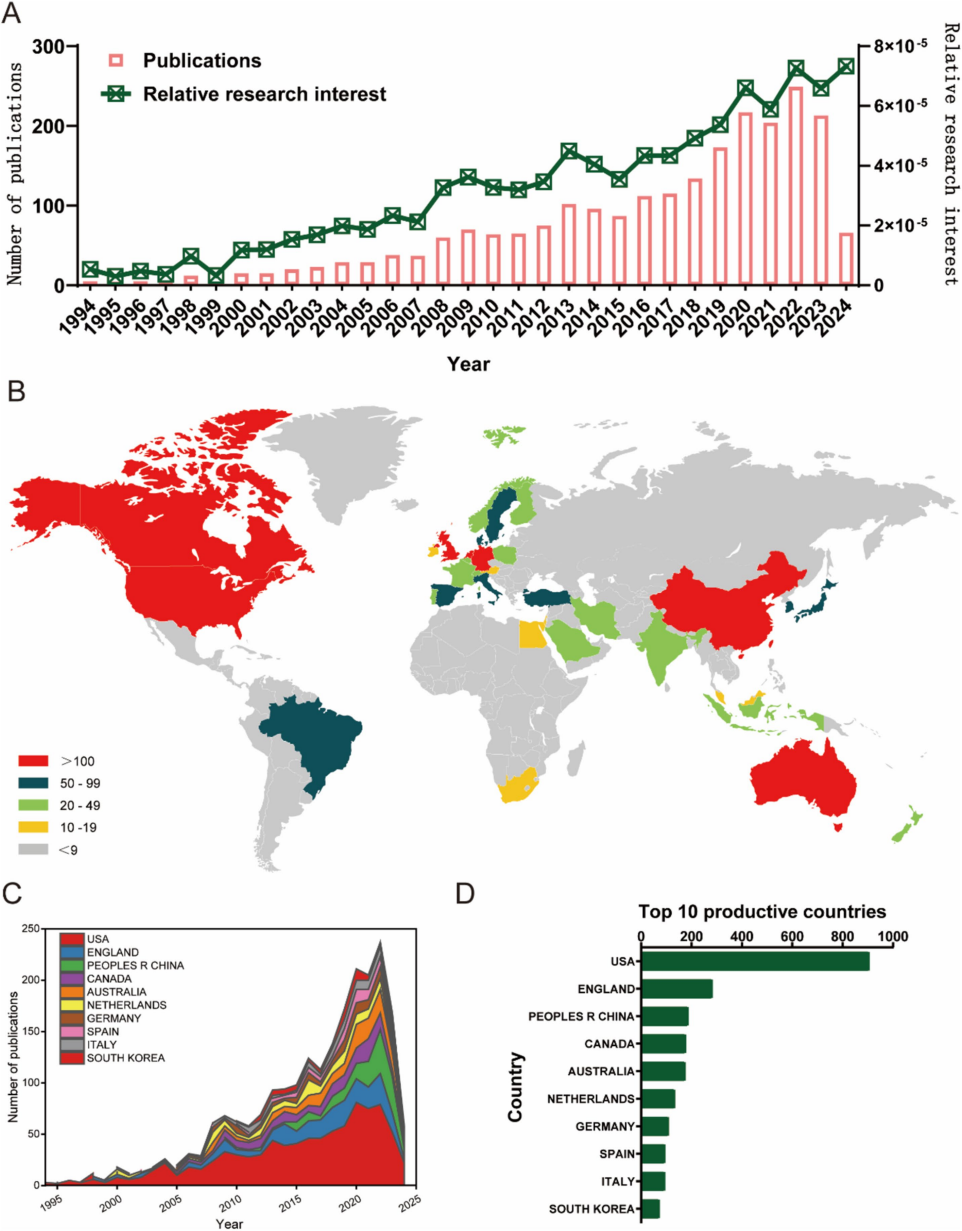
Global trends and countries/regions contributing to the research field regarding osteoarthritis with depression from 1994 to 2024. **(A)** The annual number of publications related to osteoarthritis with depression from 1994 to 2024. **(B)** A world map depicting the distribution of osteoarthritis with depression from 1994 to 2024. The total number **(C)** and annual number **(D)** of publications in the top 10 most productive countries from 1994 to 2024.

We summarized the trends in the publication volume and distribution of global literature (Figure 2). As shown in Figure 2A, from 1994 to 2007, the annual number of publications in this field did not exceed 50. There was a significant upward trend in the annual publication volume, peaking between 2020 and 2023, with each of these four years exceeding 200 publications, the highest being in 2022. The lower volume for 2024, less than 100, is due to partial data, as many articles have not yet been published. A total of 79 countries/regions worldwide have published English-language literature on OA with depression research (Figure 2B). The top 10 countries/regions by annual number of publications first exceeded 50 in 2008 and continued to grow, surpassing 200 for the first time in 2020 (Figure 2C). As shown in Figure 2D, from 1994 to 2024, the countries with the highest total number of publications were the United States (31.2%), the United Kingdom (20.8%), China (19.5%), Canada (19.4%), Australia (14.8%), the Netherlands (12%), Germany (10.5%), Spain (10.4%), and Italy (8.1%). Overall, in the past 30 years, particularly after 2008, research on OA with depression has developed rapidly and is increasingly attracting the attention of researchers.

### 3.2 Analysis of national/regional and institutional cooperation in global literature

**Figure 3:**
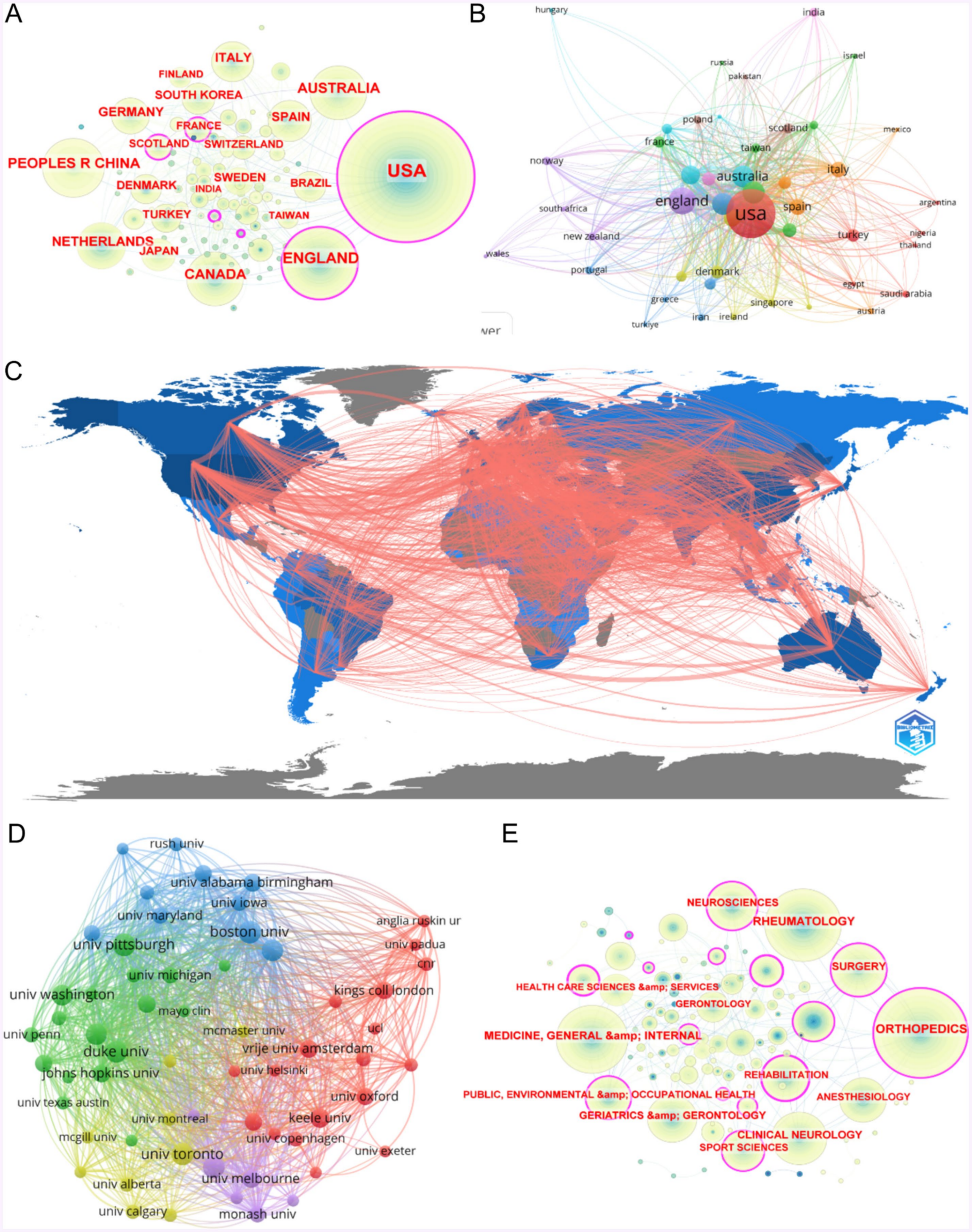
Mapping of countries/regions and institutions associated with osteoarthritis with depression. **(A)** Country/regional collaboration analysis based on CiteSpace. **(B)** Mapping of the 47-country with >5 publications. **(C)** The geographical network map based on R software. **(D)** Mapping of the 60-institution collaboration analysis. The nodes represent institutions, and the lines connect them. The number of publications grows proportionally to the size of the nodes. The lines between the nodes represent the cooperation relationship. **(E)** The research category network map of osteoarthritis with depression.

Figure 3A illustrates a country/region collaboration analysis in the field of OA with depression using CiteSpace. The lines indicate collaborations, while the size and color of the nodes represent the number of publications over different years. Countries highlighted in purple have significant influence. The United States and the United Kingdom, with the highest number of publications, are central to international collaboration. Figure 3B, created with VOSviewer, displays the collaborative relationships among 47 countries, each with more than five publications. The lines indicate collaboration, and node size represents link strength, with the strongest links being between the United States and the United Kingdom. Figure 3C is a geographical network map, offering an intuitive display of publication and collaboration in OA with depression research. North America, East Asia, and Western Europe are the primary regions conducting this research, with significant mutual collaboration.

Figure 3D presents an institutional collaboration analysis of 60 institutions, each with more than 15 publications. Lines represent collaborations, and node size corresponds to the number of publications. National contributions to this field vary significantly, but at the institutional level, top institutions contribute relatively evenly. Table 1 highlights the impact of the United States, which has the highest total citation count of 34,422 and a total link strength of 251,540, surpassing the United Kingdom (10,044 citations, 118,366 link strength) and Canada (6,950 citations, 86,904 link strength). Among institutions, Boston University leads with a total citation count of 2,989 and a total link strength of 49,789, followed by Duke University (2,381 citations, 47,194 link strength) and the University of California, San Francisco (1,380 citations, 46,835 link strength).

**Table 1:** The top 10 countries and institutions contributed to the publications.

Rank	Countries	Citations/Total link strength	Institution	Citations/Total link strength
1	USA	34,422/251540	Boston Univ	2,989/ 49,789
2	England	10,044/118366	Duke Univ	2381/47194
3	Canada	6950/86904	Univ Calif **S**an Francisco	1380/46835
4	Australia	5011/84924	Univ Pittsburgh	1922/45568
5	Netherlands	4960/69009	Univ Toronto	2412/43181
6	China	3410/58765	Univ Sydney	1688/38801
7	Germany	2899/42344	Univ Melbourne	709/36696
8	Spain	2190/42156	Harvard med Sch	840/33992
9	Italy	2201/34544	Northwestern Univ	1695/31319
10	Sweden	1946/27951	Vrije Univ Amsterdam	1177/29630

### 3.3 Analysis of research fields of global literature

Figure 3E presents the research category network map for the field of OA (OA) with depression. In this map, nodes represent research categories, with the size of each node indicating the number of articles within that category. The lines between nodes represent interdisciplinary collaboration. It can be observed that Orthopedics has the greatest influence in the research on OA with depression. The research types are not homogeneous, which is some evidence that research on OA and depression involves multifaceted studies and is valued by multiple fields.

### 3.4 Analysis of authors and journals of global literature

**Figure 4:**
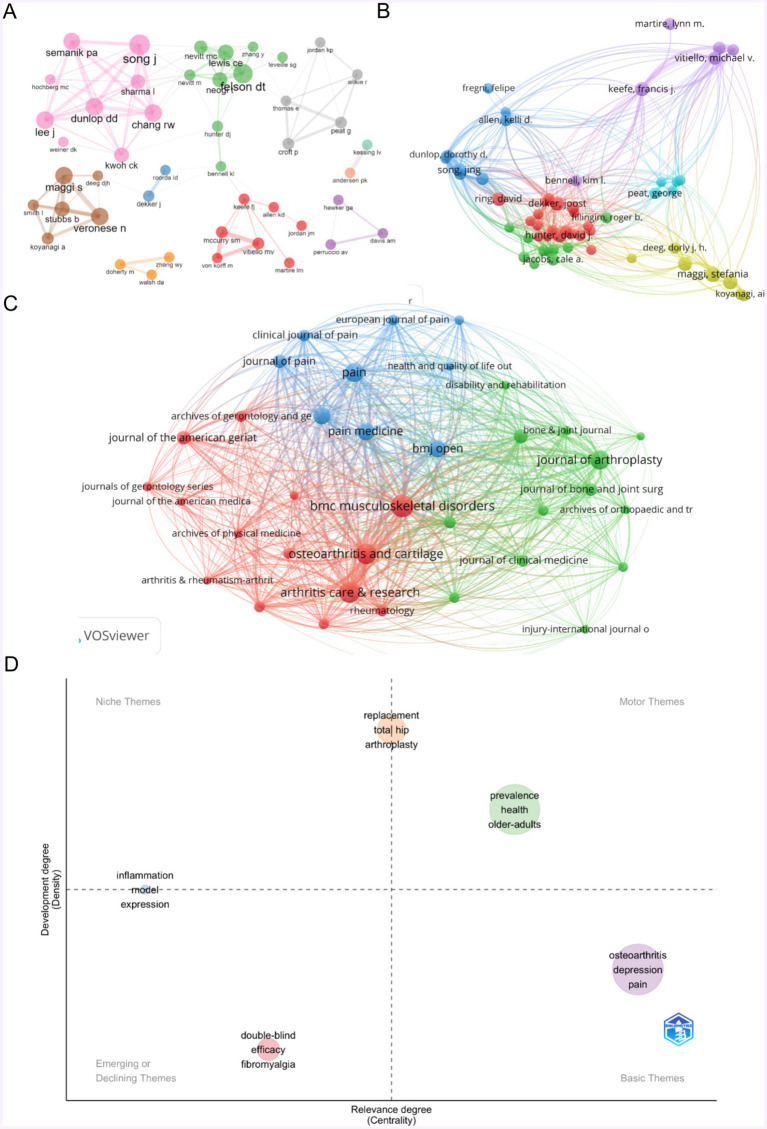
Mapping of authors and journals in studies on osteoarthritis with depression. **(A)** Collaboration analysis of the authors network based on R software. **(B)** 57-author with publications more than 8. **(C)** Journals collaboration analysis. **(D)** Strategic theme map based on R software.

Our literature search identified a total of 11,241 authors. Figure 4A shows an author collaboration analysis using R bibliometrix software, where nodes represent authors, the size of the nodes indicates the number of publications, and lines represent collaborative relationships. Figure 4B, created with VOSviewer, depicts the collaboration among 57 authors who have been cited more than eight times. Table 2 lists the top 10 authors in the field of OA with depression based on the number of publications. Maggi Stefania ranks first with 19 papers and a total link strength of 15,003. Song Jing is second with 14 papers and a total link strength of 14,622, followed by Sharma Leena with 11 papers and a total link strength of 13,549. The contributions among these leading authors are notably similar.

**Table 2:** The top 10 active authors with the most publications on this area.

Rank	Highly published authors	Article counts	Total link strength
1	Maggi, Stefania	19	15,003
2	Song, Jing	14	14,622
3	Sharma, Leena	11	13,549
4	Keefe, Francis J.	16	13,273
5	Veronese, Nicola	17	12,543
6	Dekker, Joost	16	11,454
7	Stubbs, Brendon	16	11,020
8	Allen, Kelli D.	12	10,972
9	Bennell, Kim	11	10,483
10	Kwoh, C. Kent	13	10,331

The top 10 journals publishing papers on OA with depression are listed in Table 3. *BMC Musculoskeletal Disorders* (Impact Factor = 2.2) ranks first with 71 articles and a total of 1,395 citations. *Arthritis Care & Research* (Impact Factor = 3.7) ranks second with 60 articles and 1,513 citations. *Osteoarthritis and Cartilage* (Impact Factor = 7.2) ranks third with 58 articles and 3,340 citations. Figure 4C shows the journal collaboration analysis, indicating that *BMC Musculoskeletal Disorders*, with the highest number of publications, has the greatest impact, with a link strength of 16,585. *Arthritis Care & Research*, *Osteoarthritis and Cartilage,* and *Pain* also hold significant positions in the collaboration network. Figure 4D, created with R bibliometrix software, presents a strategic theme map in the field of OA with depression. In this map, nodes represent popular research topics, the horizontal axis represents the relevance degree (centrality), and the vertical axis represents the development degree (density). Nodes in the upper right position (first quadrant) are highly relevant to the field of OA with depression and are studied more frequently and in greater depth. The three prominent themes identified are replacement total hip arthroplasty, association risk population, and depression prevalence health.

**Table 3:** The top 10 journal published most articles.

Rank	Journal	Article counts	Citations	Impact factor (IF, 2023)
1	BMC Musculoskeletal Disorders	71	1,395	2.2
2	Arthritis Care & Research	60	1,513	3.7
3	Osteoarthritis and Cartilage	58	3,340	7.2
4	Pain	49	3,572	5.9
5	Journal of Arthroplasty	51	1,062	3.4
6	Pain Medicine	42	886	2.9
7	BMJ Open	40	388	2.4
8	Clinical Orthopedics and Related Research	27	1,485	4.2
9	Rheumatology	17	999	4.7
10	Plos One	41	1,269	2.9

### 3.5 Analysis of reference of global literature

**Figure 5:**
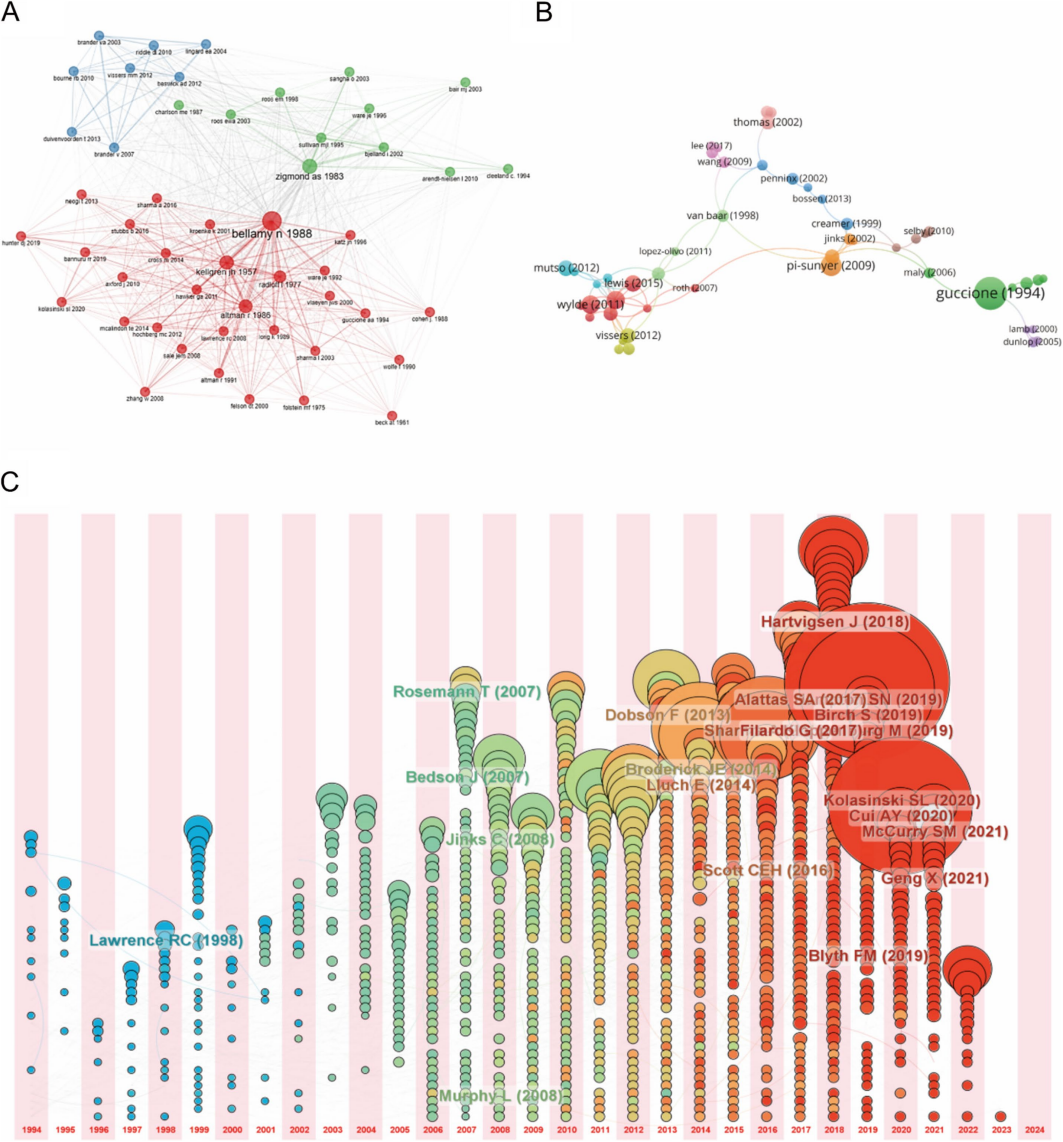
Mapping of cited references in studies on osteoarthritis with depression. **(A)** Mapping of the reference co-citation network in this field. **(B)** Mapping of the 147-refencence with citation more than 100 in this field. **(C)** Reference growth and decline, the larger the node area, the more important the reference in a given year.

Using VOSviewer, we mapped the co-citation network of references in the field of OA with depression (Figure 5A). Bellamy N’s 1988 article, titled “Validation study of WOMAC: A health status instrument for measuring clinically important patient relevant outcomes to antirheumatic drug therapy in patients with OA of the hip or knee,” published in *The Journal of Rheumatology*, has been cited 260 times and holds a central position in the network. From the 2,431 retrieved articles, we selected 147 that were cited more than 100 times to create the high-citation analysis shown in Figure 5B.

Table 4 lists the top 10 co-cited references, with Bellamy’s 1988 article leading the list. The second most co-cited reference is “Radiological assessment of osteoarthrosis” by Kellgren J H, published in *Annals of the Rheumatic Diseases*. The third is “Development of criteria for the classification and reporting of osteoarthritis: classification of osteoarthritis of the knee” by Altman R, published in *Arthritis & Rheumatism*. Figure 5C provides a timeline visualization of the references, showing the most frequently cited references from 1994 to 2024, with larger node areas indicating more important references in each year.

**Table 4:** The top 10 co-cited reference (CR) in the field.

Rank	Citation	First author	Journal	Title
1	260	Bellamy N	The Journal of Rheumatology	Validation study of WOMAC: a health status instrument for measuring clinically important patient relevant outcomes to antirheumatic drug therapy in patients with osteoarthritis of the hip or knee.
2	175	Kellgren J H	Annals of the Rheumatic Diseases	Radiological assessment of osteoarthrosis.
3	164	Altman R	Arthritis & Rheumatism	Development of criteria for the classification and reporting of osteoarthritis: classification of osteoarthritis of the knee.
4	211	A S Zigmond	Acta Psychiatrica Scandinavica	The hospital anxiety and depression scale
5	130	Sullivan, Michael J. L.	Psychological Assessment	The Pain Catastrophizing Scale: Development and validation.
6	183	Radloff	Applied Psychological Measurement	The CES-D Scale: A Self-Report Depression Scale for Research in the General Population
7	65	Andrew David Beswick	BMJ Open	What proportion of patients report long-term pain after total hip or knee replacement for osteoarthritis? A systematic review of prospective studies in unselected patients
8	76	Marita Cross	Annals of the Rheumatic Diseases	The global burden of hip and knee osteoarthritis: estimates from the global burden of disease 2010 study
9	51	Victoria Brander	Clinical Orthopedics and Related Research	Pain and depression influence outcome 5 years after knee replacement surgery
10	71	Gillian A Hawker	Arthritis Care & Research	A longitudinal study to explain the pain-depression link in older adults with osteoarthritis

### 3.6 Keyword analysis

**Figure 6:**
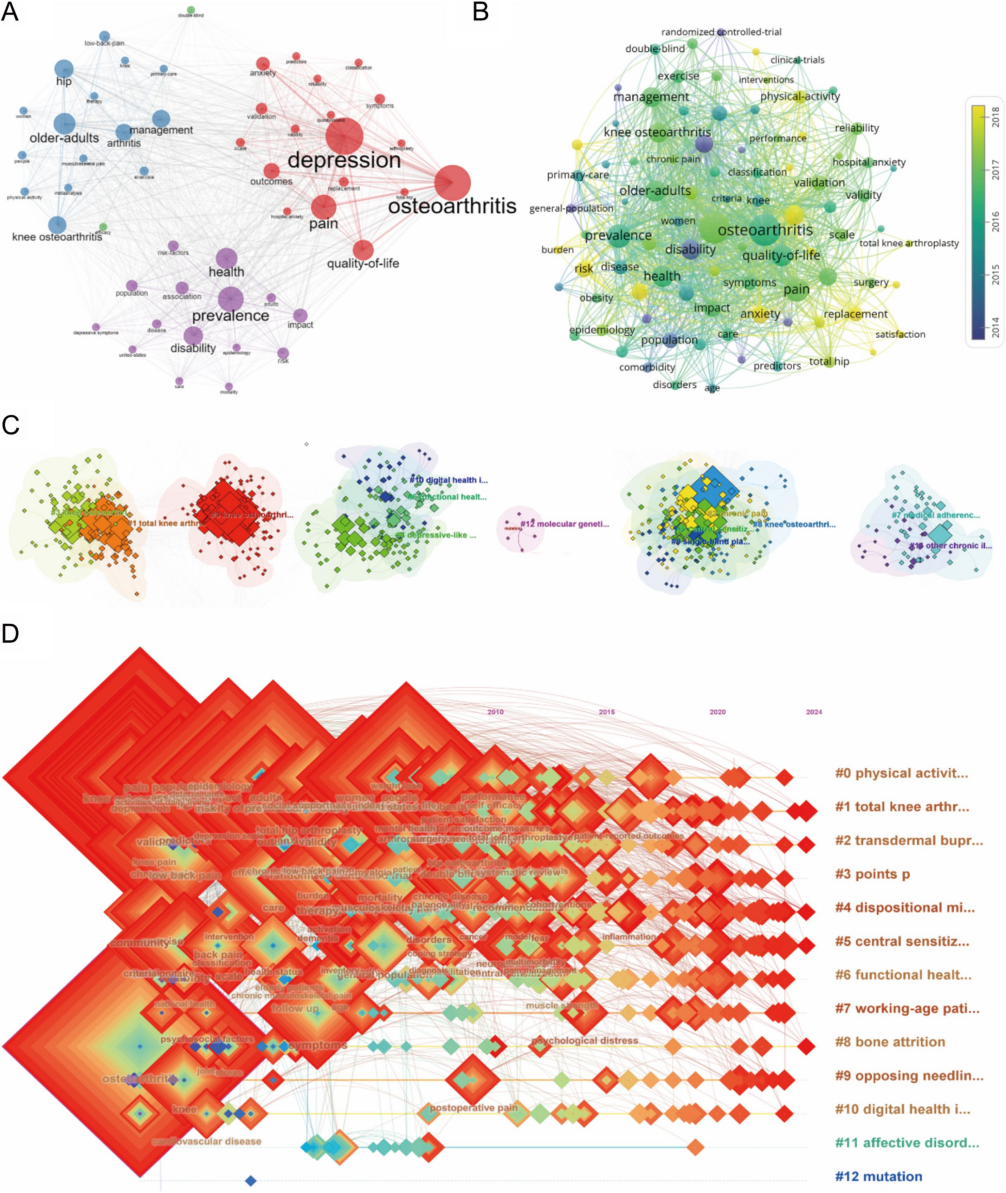
Keyword analysis. **(A)** Co-occurrence analysis of the keyword network based on R software. **(B)** Distribution of keywords according to average publication year (blue: earlier, yellow: later) by VOSviewer. **(C)** Clustering analysis of the keyword network based on CiteSpace. **(D)** Keyword timeline visualization from 1994 to 2024 by CiteSpace.

**Figure 7:**
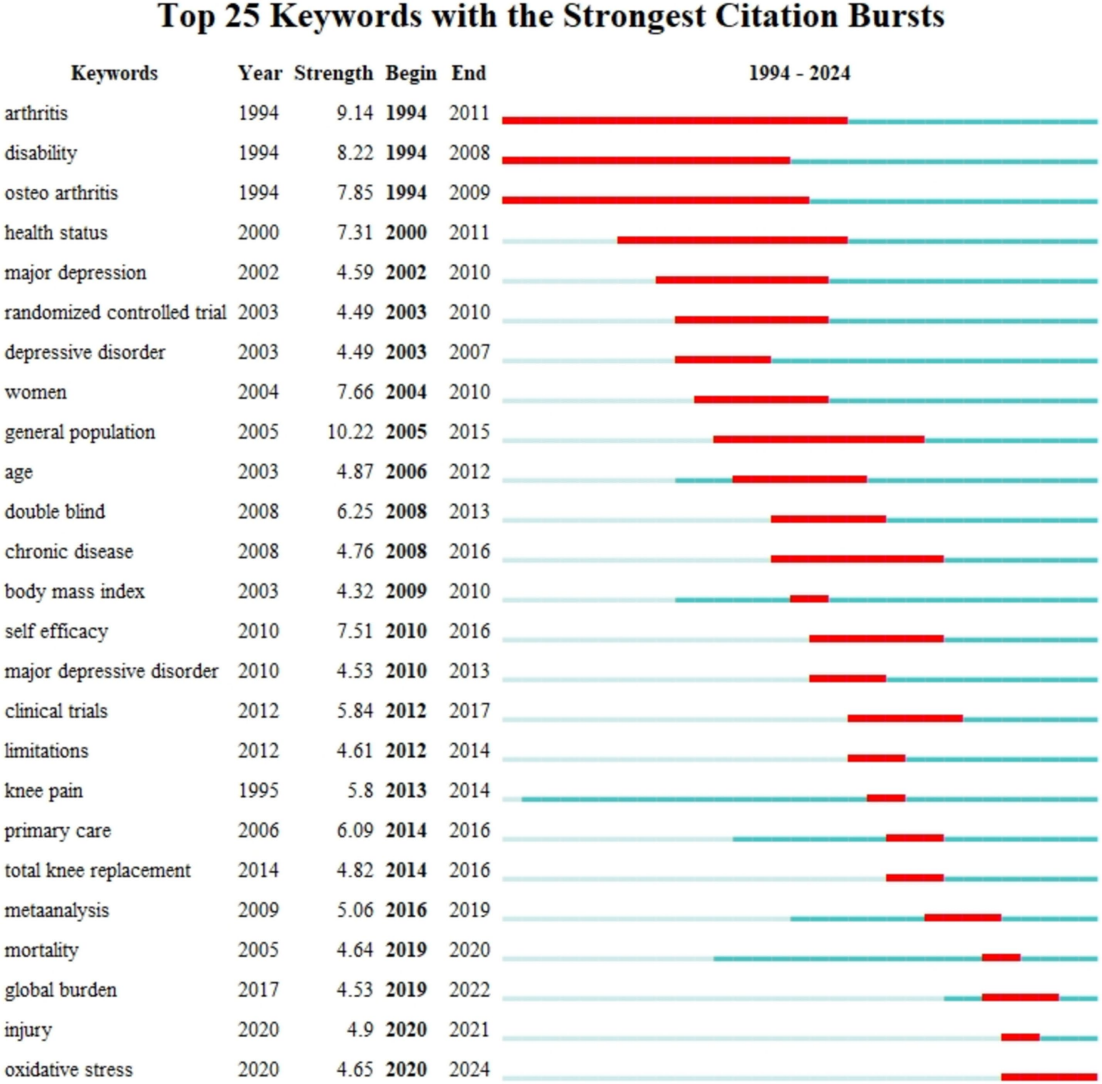
Top 25 keywords with the strongest citation bursts of publications related to osteoarthritis with depression.

A total of 4,127 keywords were identified from the retrieved articles, with 90 keywords appearing more than 30 times. We used VOSviewer to visualize these keywords in a network (Figure 6A). Lines in the visualization represent the co-occurrence of keywords, with “depression” and “osteoarthritis” showing 605 and 611 co-occurrences, respectively, far surpassing other keywords. Based on this, we visualized the average publication year of the keywords (Figure 6B). Subsequently, we performed further clustering based on the characteristics of the keywords (Figure 6C), identifying 13 clusters, including knee osteoarthritis (cluster 0), total knee arthroplasty (cluster 1), and chronic pain (cluster 2), among others. Figure 6D is a timeline visualization of the keywords, illustrating the evolution of hotspot keyword clusters in the field of OA with depression from 1994 to 2024. Lines represent the co-occurrence of keyword clusters. In the early period (1994–2000), keywords were fewer and more concentrated, with hotspots like “pain” and “osteoarthritis.” In the middle period (2001–2009), the number of keywords greatly increased, but the research still had a clear focus, revolving around “total knee arthroplasty” and “symptoms.” In the recent period (2010–2024), keywords have become more dispersed and refined, indicating that the research has reached a significant depth. Moreover, a citation burst refers to a frequency surge of keywords. CiteSpace enables burst detection across several categories: (1) Single or multi-word phrases found in titles, abstracts, or other sections of a publication; (2) The citation counts of referenced works over time; (3) The frequency of keyword occurrences over time; and (4) The number of publications produced by an author, institution, or country (Kleinberg, 2002). Therefore, as shown in Figure 7, the top 25 keywords with the strongest citation bursts. In the last column of this figure, the red bars indicate that the strongest citation bursts occurred during this period, and the blue bars represent the period that covers burst detection. The top three keywords, “arthritis,” “disability,” and “osteoarthritis,” have very long durations, each lasting over 15 years.

## 4 Discussion

### 4.1 Research status

This research applied CiteSpace, VOSviewer, and R bibliometrix software to conduct a bibliometric analysis of published articles relating to studies about OA and depression. The number of publications in this field has shown a consistent upward trend globally, with a significant surge beginning in 2015. Most of these publications originate from the United States and England, together contributing over 50% of the total. Notably, seven of the top ten contributing institutions are from the United States, and two are from Australia, highlighting the importance of world-class research institutions and substantial research investment to enhance a country’s academic standing. Among the top ten journals publishing research on OA and depression, only two journals boast impact factors greater than 5: *Osteoarthritis and Cartilage* (ranked 3rd) and *Pain* (ranked 4th).

Additionally, only *Arthritis Care & Research* (ranked 2nd), *BMJ Open* (ranked 7th), *Clinical Orthopedics and Related Research* (ranked 8th), and *Plos One* (ranked 10th) were established within the past 20 years. Regarding access methods, three journals—*BMC Musculoskeletal Disorders* (ranked 1st), *BMJ Open* (ranked 7th), and *Plos One* (ranked 10th)—are open access. It is evident that authors in the field of OA with depression tend to favor newly established, open-access journals over traditional ones, despite the latter’s higher impact factors. This preference may stem from the broader audience and faster review and publication processes offered by these journals. *BMJ Open* is particularly noteworthy, having been founded in 2011, making it the most recent among the top ten journals. Despite its recent establishment, it has published eight articles in this field, establishing itself as a significant platform for disseminating research on OA with depression. Hence, submitting to such journals, as listed in Table 3, is recommended.

In the research area of OA and depression, rheumatology and orthopedic surgery are pivotal, which explains why the top ten journals are related to orthopedics. Among the top thirteen well-represented research categories, five fall within rheumatology and orthopedic surgery, with others related to neurosciences and public/environmental science. This reflects the frequent interdisciplinary interactions inherent in this field, underscoring the cross-disciplinary nature of research on orthopedics and healthcare sciences. Overlay visualization analysis reveals that research is predominantly concentrated in the fields of orthopedics, rheumatology, and healthcare sciences (Table 5).

**Table 5:** The top 10 cited articles in the field.

Rank	Total citation	Title	First author	Journal	Main point
1	3,968	Global, regional, and national incidence, prevalence, and years lived with disability for 301 acute and chronic diseases and injuries in 188 countries, 1990–2013: a systematic analysis for the Global Burden of Disease Study 2013.	Theo Vos	The Lancet	Provide a comprehensive assessment of the burden of a wide range of diseases and injuries worldwide over 23 years, highlighting trends and changes in health across different regions and countries.
2	1,207	The effects of specific medical conditions on the functional limitations of elders in the Framingham Study.	AndrewA. Guccione,	American journal of Public Health	Specific medical conditions such as stroke, depressive symptomatology, hip fracture, knee osteoarthritis, and heart disease significantly contribute to physical disability in noninstitutionalized elderly men and women.
3	1,205	Effect of duloxetine on pain, function, and quality of life among patients with chemotherapy-induced painful peripheral neuropathy: a randomized clinical trial.	Ellen M. Lavoie Smith	JAMA	Duloxetine significantly reduces pain, improves function, and enhances quality of life in patients with chemotherapy-induced painful peripheral neuropathy compared to placebo.
4	1,190	Recurrent concussion and risk of depression in retired professional football players.	Kevin M. Guskiewicz	Clinically Relevant	Recurrent concussions in retired professional football players are significantly associated with an increased risk of being diagnosed with clinical depression, with the likelihood of depression rising with the number of concussions sustained.
5	651	The medical risks of obesity.	Xavier Pi-Sunyer	Postgraduate Medicine	Obesity significantly increases the risk of various serious health conditions, including cardiovascular diseases, diabetes, certain cancers, sleep apnea, and joint diseases, ultimately contributing to increased morbidity and mortality.
6	641	Test–retest reliability of Quantitative Sensory Testing in knee osteoarthritis and healthy participants.	V. Wylde	Osteoarthritis and Cartilage	The study found that pressure pain thresholds (PPTs) showed high test–retest reliability in both knee osteoarthritis patients and healthy participants, supporting the use of PPTs in assessing pain perception in osteoarthritis research.
7	554	Predicting total knee replacement pain: a prospective, observational study	Victoria A. Brander	Clinical Orthopedics and Related Research	Preoperative pain, depression, and anxiety are significant predictors of postoperative pain, with pain levels generally declining over time but about 13% of patients still experiencing moderate to severe pain one year after surgery despite no clinical or radiographic abnormalities.
8	541	Predictors of persistent pain after total knee arthroplasty: a systematic review and meta-analysis.	G. N. Lewis	British Journal of Anesthesia	Preoperative pain, catastrophizing, mental health, and pain at other sites are the strongest independent predictors of persistent pain after total knee arthroplasty.
9	439	The association between chronic pain and obesity.	Akiko Okifuji	Journal of Pain Research	Obesity and chronic pain are often comorbid conditions, with multiple factors such as mechanical stress, chemical mediators, and lifestyle contributing to their interrelationship.
10	437	Abnormalities in hippocampal functioning with persistent Pain.	Amelia A. Mutso	The Journal of Neuroscience	Persistent pain leads to hippocampal abnormalities, including reduced neurogenesis, altered synaptic plasticity, and decreased hippocampal volume, which are associated with learning, emotional, and cognitive deficits in both animal models and chronic pain patients.

Multiple collaboration analyses indicate that collaborations among authors or institutions in this field are often limited to within the same country, highlighting the need for more international cooperation. The most co-cited paper is “Validation study of WOMAC: A health status instrument for measuring clinically important patient relevant outcomes to antirheumatic drug therapy in patients with OA of the hip or knee,” published in 1988 in *The Journal of Rheumatology*. The most cited research article in this field is “Global, regional, and national incidence, prevalence, and years lived with disability for 301 acute and chronic diseases and injuries in 188 countries, 1990–2013: A systematic analysis for the Global Burden of Disease Study 2013,” published in 2015 in *The Lancet*. These highly cited articles typically focus on themes such as chronic disease burden and OA treatment research. Cluster analysis of the references corroborates these observations, with popular topics including arthroplasty, population, double-blind, randomized controlled trial, depression prevalence, pain, and quality of life.

### 4.2 Frontiers in the future

**Figure 8:**
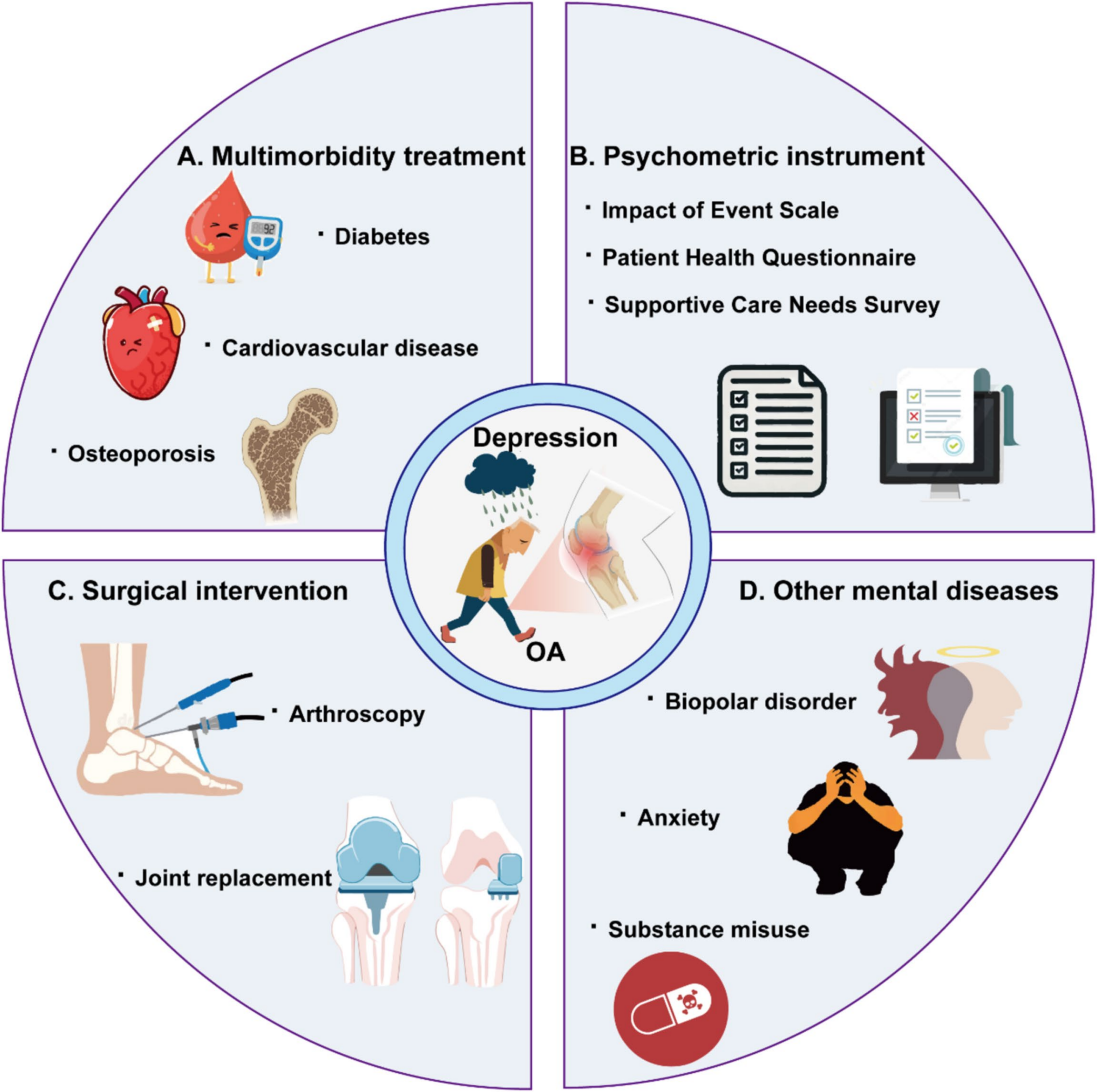
Schematic illustration of research frontiers in the future about osteoarthritis with depression.

Based on the keywords with the highest citation bursts that last till 2024, we forecast frontiers as follows (Figure 8):

#### 4.2.1 Treating depression in patients with osteoarthritis with multimorbidity

*Multimorbidity*, defined as concurrently having at least two chronic conditions, is more common, particularly among the elderly as the population ages (Salive, 2013). It is especially prevalent in patients with OA, who often experience multiple chronic conditions (Kővári et al., 2020). This situation is linked to lower levels of physical activity, underscoring the importance of partner support to maintain an active lifestyle (Yu et al., 2020). Among the chronic conditions studied were OA and depression, which are prevalent in this population. Hughes et al. (2013) highlighted the challenges of applying clinical guidelines, such as those for OA and depression, to patients with multimorbidity. The study emphasized the necessity of patient-centered care and compliance with treatment recommendations in managing comorbid conditions. Wang et al. (2009) focused on the effectiveness of Tai Chi in treating knee OA symptoms, suggesting it may also benefit patients with both OA and depression. Similarly, Knowles et al. (2015) evaluated an integrated collaborative care model for managing depression in patients with multimorbidity, emphasizing the need for a comprehensive approach to address mental health issues in this population. Salisbury et al. (2018) conducted a pragmatic cluster-randomized trial of a patient-centered care model, known as the 3D approach, for patients with multimorbidity. The study aimed to improve health-related quality of life, which is crucial for individuals with OA and depression. Furthermore, Porter et al. (2021) explored the integration of patient-reported outcome measures (PROMs) into routine nurse-led primary care for patients with multimorbidity, highlighting the importance of personalized care and feedback in managing multiple chronic conditions. Overall, the literature suggests that treating depression in patients with OA and multimorbidity requires a comprehensive and patient-centered approach that considers the unique challenges and needs of individuals with multiple chronic conditions. Further research, as recommended by Xu et al., is needed to provide more definitive evidence on multimorbidity management strategies.

#### 4.2.2 Psychometric properties of instruments for assessing depression and anxiety in patients with osteoarthritis

Various studies have examined the psychometric properties of instruments for assessing depression and anxiety in different patient populations. Mystakidou et al. (2007) found that the Impact of Event Scale is a valid research tool for assessing the impact of cancer diagnosis in advanced cancer patients, with construct validity supported by correlations with anxiety and depression. Similarly, Navinés et al. (2012) evaluated the Patient Health Questionnaire as a screening instrument for depression and anxiety in chronic hepatitis C patients, demonstrating reliability and validity in this population. In the context of cancer patients, Kotronoulas et al. (2011) assessed the psychometric properties of the Greek Pittsburgh Sleep Quality Index, highlighting its reliability and validity in measuring sleep quality in patients undergoing chemotherapy. Moreover, Paiva et al. evaluated the European Organization for Research and Treatment of Cancer Core Quality of Life Questionnaire in a Brazilian cancer patient cohort, confirming its validity across different educational levels (Paiva et al., 2014). Furthermore, the study by Wardenaar et al. (2010) introduced a short adaptation of the Mood and Anxiety Symptoms Questionnaire, the MASQ-D30, which demonstrated a clearer factor structure and suitability for large-scale psychopathology research. Sklenarova et al. (2015) validated the German version of the Supportive Care Needs Survey for Partners and Caregivers, emphasizing its utility in assessing caregivers’ needs throughout the disease trajectory. While these studies focus on various patient populations, such as cancer and hepatitis C patients, the psychometric properties of instruments for assessing depression and anxiety in patients with OA remain to be explored. Future research could adapt and validate existing instruments, such as the MASQ-D30 or the Patient Health Questionnaire, for use in OA patients to improve the assessment of these common comorbidities in this population.

#### 4.2.3 Depression or anxiety in patients with surgical intervention

Depression and anxiety are common comorbidities in patients undergoing surgical interventions for various medical conditions. In a study by Marks (2009), it was found that these mental health issues impact the disability experienced by patients with hip OA both before and after total hip replacement surgery. This highlights the need to address mental health in patients with OA undergoing surgery. In a study by Kulu et al. (2020), the anxiety-depression and somatization levels of patients scheduled for surgery due to OA were evaluated. Their findings highlight the emotional challenges faced by these patients, emphasizing the importance of interventions targeting anxiety and depression. Furthermore, the study by Luebbert et al. (2001) demonstrated the effectiveness of relaxation training in improving emotional adjustment variables such as depression and anxiety in patients undergoing non-surgical cancer treatment. While this study focused on cancer patients, its findings suggest that similar interventions could benefit OA patients undergoing surgery by alleviating emotional distress. In conclusion, addressing depression and anxiety in patients with OA undergoing surgical interventions is crucial for improving patient outcomes and overall well-being. Interventions such as relaxation training and cognitive-behavioral therapy may play a significant role in reducing emotional distress and improving the surgical experience for these patients. Further research, such as the study proposed by Kaynar et al. (2023) on digital cognitive behavioral intervention for patients undergoing hip and knee arthroplasty surgery, is needed to explore the impact of such interventions on preoperative mood disorders and postoperative outcomes in this patient population.

#### 4.2.4 Other mental diseases in patients with osteoarthritis

Research has shown a significant association between psychiatric disorders and OA in patients. Patients with affective psychoses, such as depression and bipolar disorders, have been found to have an increased risk of developing OA (Huang et al., 2016). Additionally, individuals with RA have a higher risk of developing bipolar disorder compared to those without RA, with a pooled relative risk of 2.06 (Charoenngam et al., 2019). Furthermore, comorbid medical illnesses have been observed in patients with bipolar disorder, with those diagnosed with bipolar II disorder being more likely to have conditions such as gastric ulcers, heart disease, Parkinson’s disease, and rheumatoid arthritis (Forty et al., 2014). A study also found an increased risk of developing Parkinson’s disease in patients with bipolar disorder episodes, as well as those with OA or diabetes (Nilsson et al., 2001). The impact of RA, an autoimmune disease, on patient health has wider implications regarding health policy and costs, highlighting the importance of addressing comorbidities in these patients (Hsu et al., 2014). While S-Adenosyl-L-Methionine (SAMe) has been primarily studied for depression, OA, and liver diseases, it is important to consider its potential effects on individuals with bipolar disorder, as it is characterized by mood swings (Sharma et al., 2017). In conclusion, the relationship between bipolar disorder and OA in patients is complex and warrants further investigation to better understand the underlying mechanisms and implications for patient care. It is crucial for healthcare providers to consider comorbidities and tailor treatment plans accordingly to improve patient outcomes.

## 5 Conclusion

Using the WOSCC database, we applied bibliometric tools to analyze articles related to OA and depressive disorders from 1994 to 2024. Our goal was to determine publication patterns, identify key contributors, and highlight recent research trends. These insights can guide investigators in understanding the current state of research and identifying new directions for future studies. Our analysis shows a steady increase in annual productivity over the past 15 years, a trend that is likely to continue. Most publications originated from the United States, with Boston University and Stefania Maggi being the most productive institution and author, respectively. The top four research frontiers are “Treating depression in osteoarthritis patients with multimorbidity,” “Psychometric properties of instruments for assessing depression and anxiety in osteoarthritis patients,” “Depression or anxiety in patients with surgical intervention,” and “Other mental diseases in osteoarthritis patients.” We anticipate that our research findings will serve as a roadmap for scholars, guiding them toward promising avenues of exploration. Additionally, these insights will prove invaluable to policymakers and administrators, facilitating informed decision-making and fostering continued progress in this vital field of study.

## Data Availability

The raw data supporting the conclusions of this article will be made available by the authors, without undue reservation.
