# Supplementary material for: Osteoarthritis with depression: mapping publication status and exploring hotspots
Source: Front Psychol. 2024 Oct 24;15:1457625. doi: 10.3389/fpsyg.2024.1457625 (PMC11540689; doi:10.3389/fpsyg.2024.1457625)

**Figure 3A: Country/regional collaboration analysis based on Citespace**


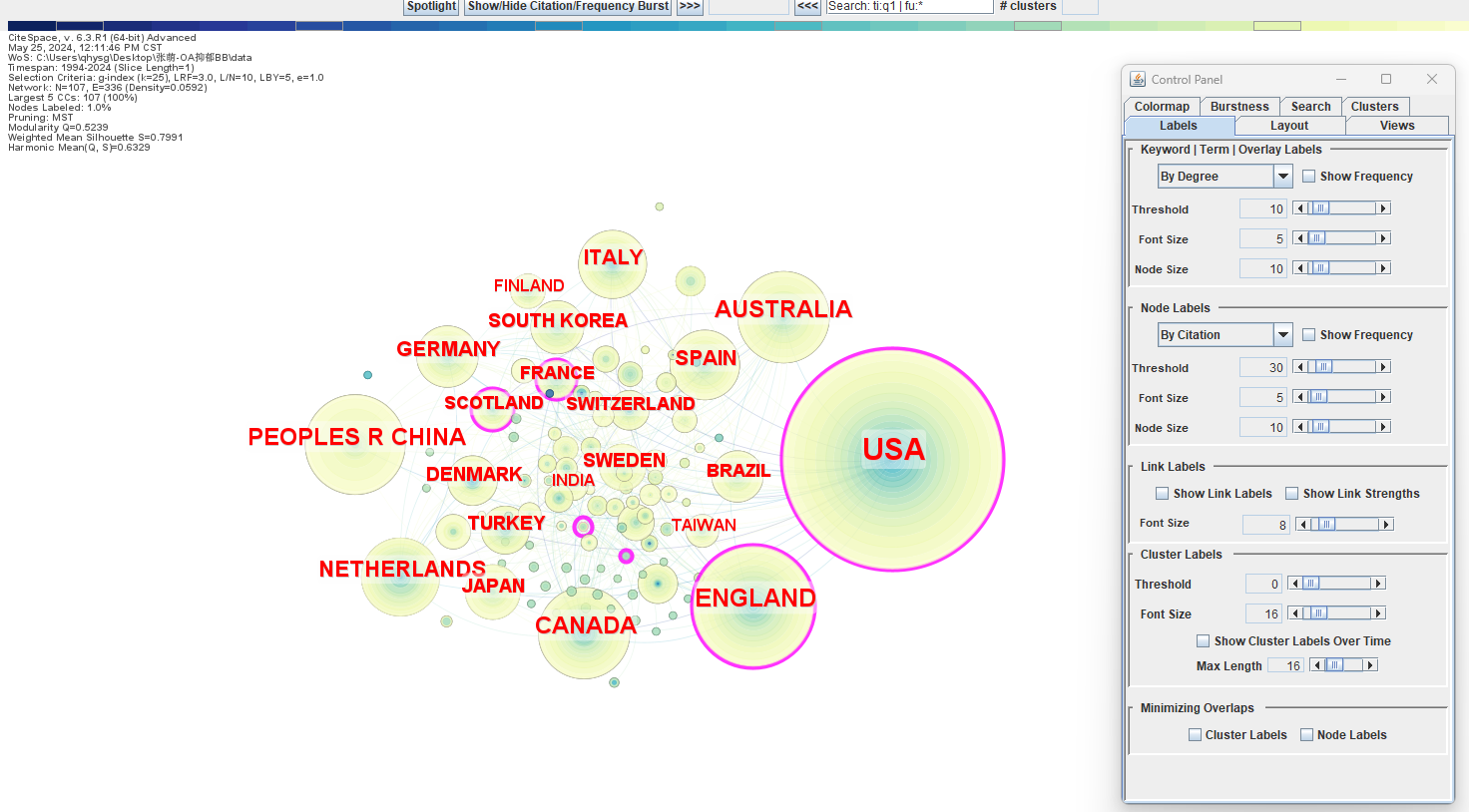


**Figure 3B: Mapping of the 47-country with >5 publications.**


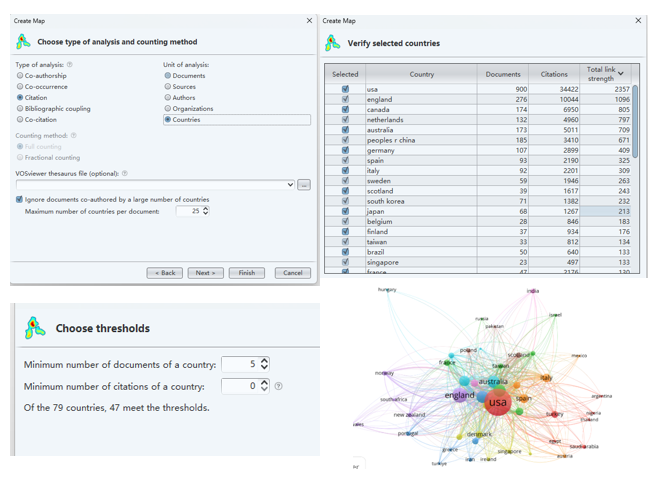


**Figure 3C: The geographical network map based on R bibliometrix.**


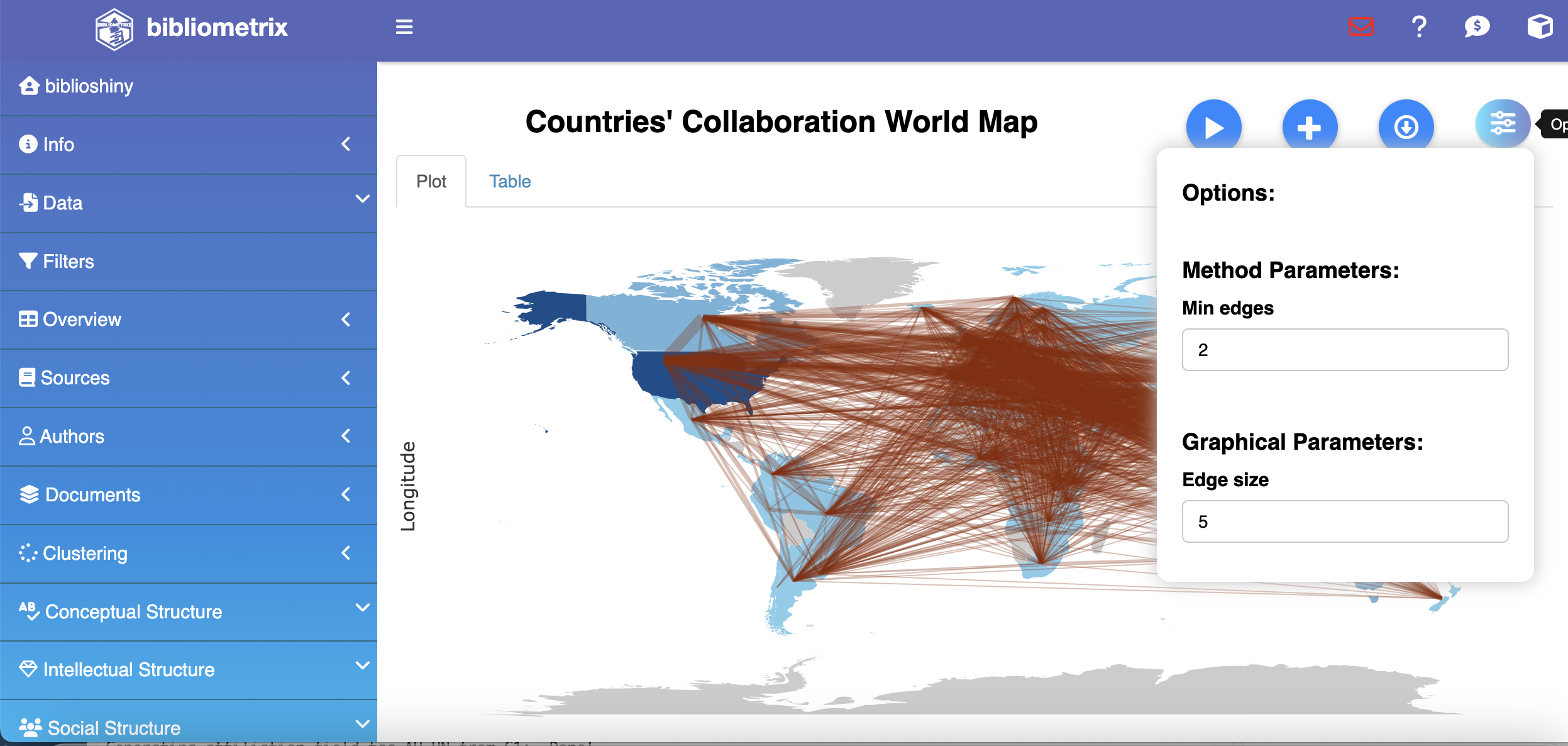


**Figure 3D: Mapping of the 60-institution collaboration analysis.**


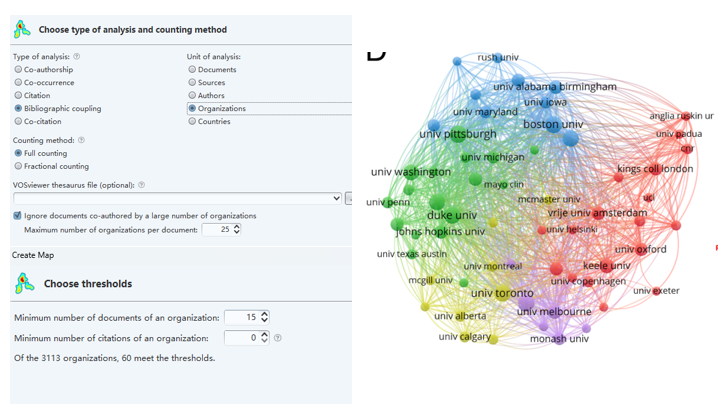


**Figure 3E: The research category network map of osteoarthritis with depression.**

**
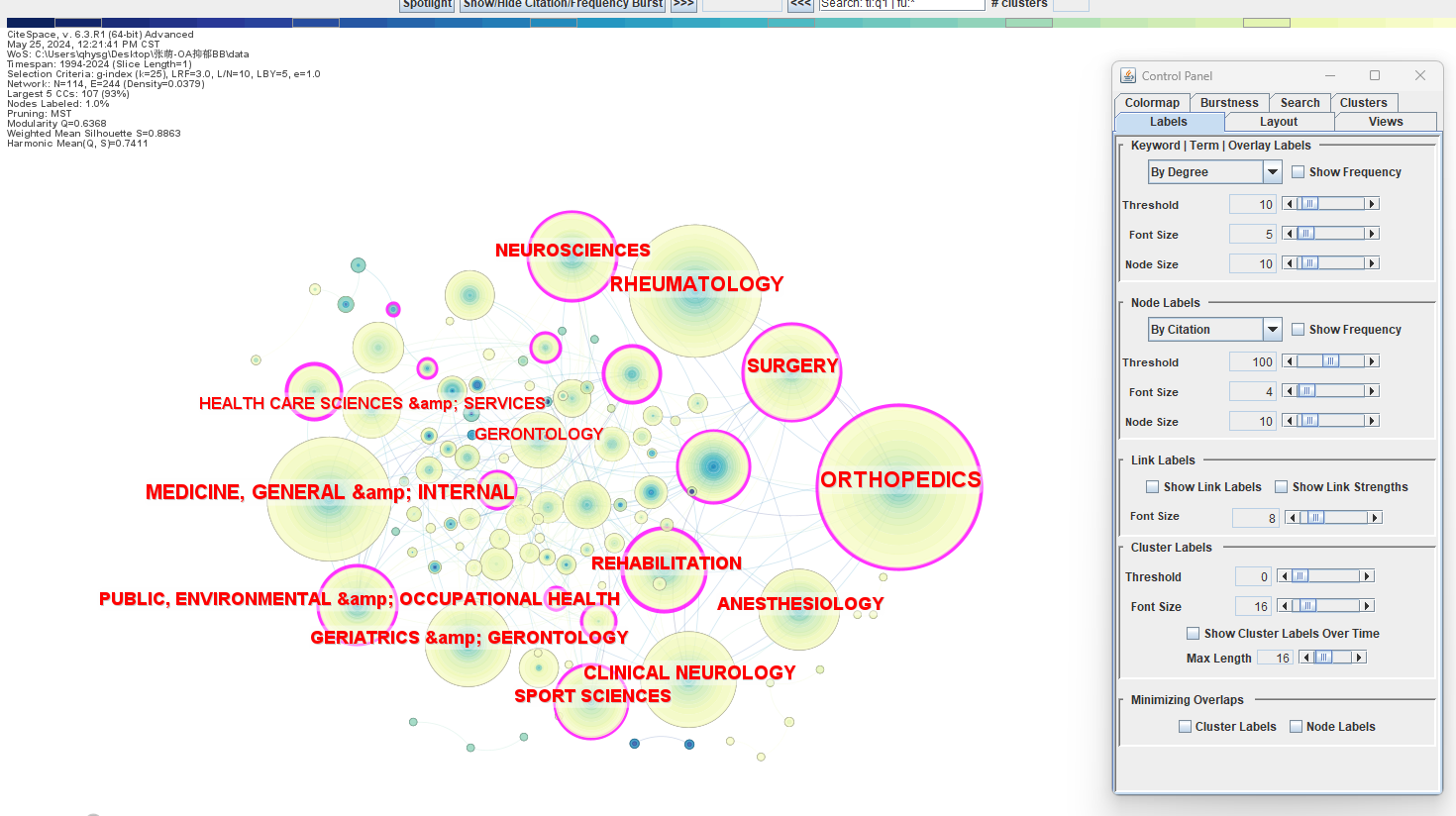
**

**Figure 4A: Collaboration analysis of the authors network based on R software.**


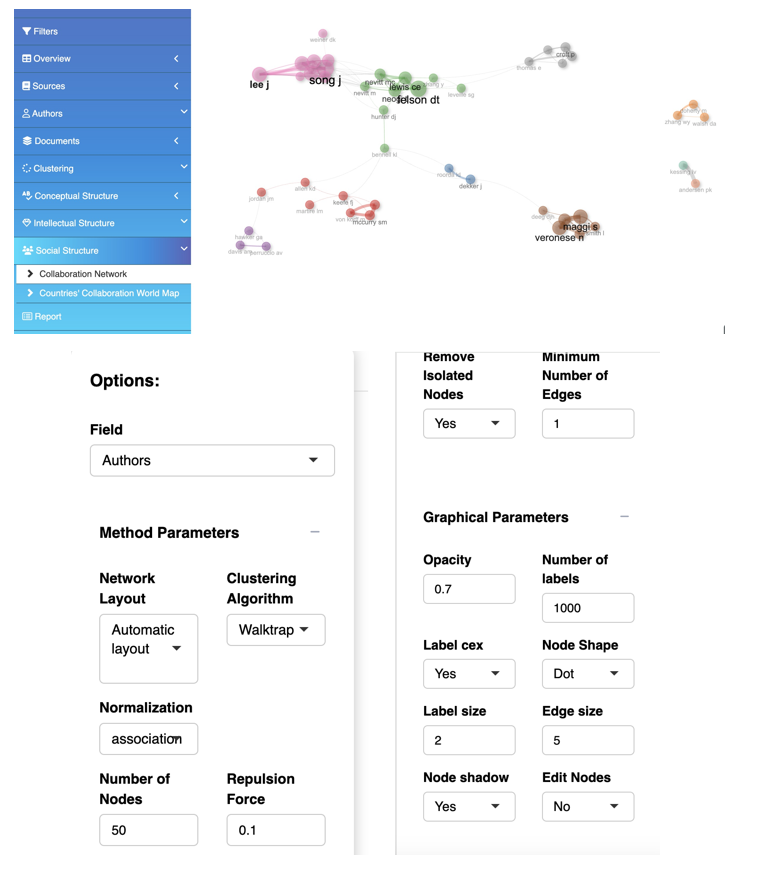


**Figure 4B: 57-author with publications more than 8.**


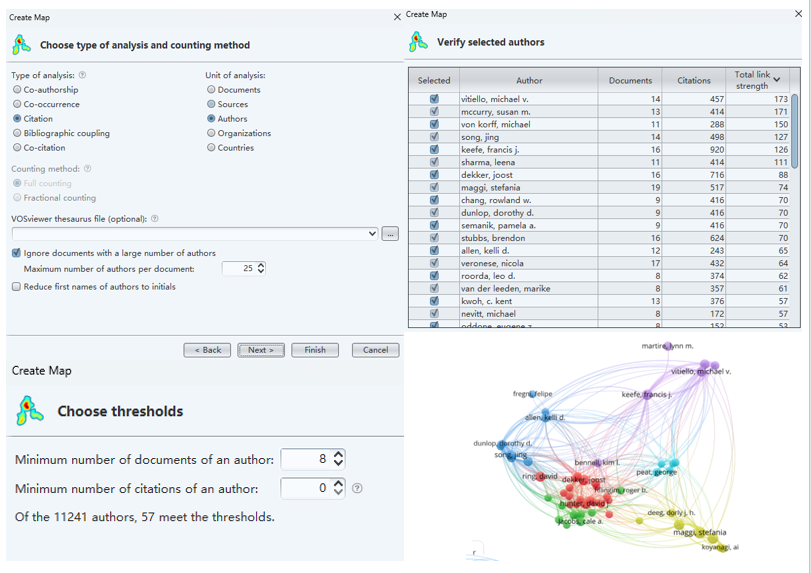


**Figure 4C: Journals collaboration analysis.**


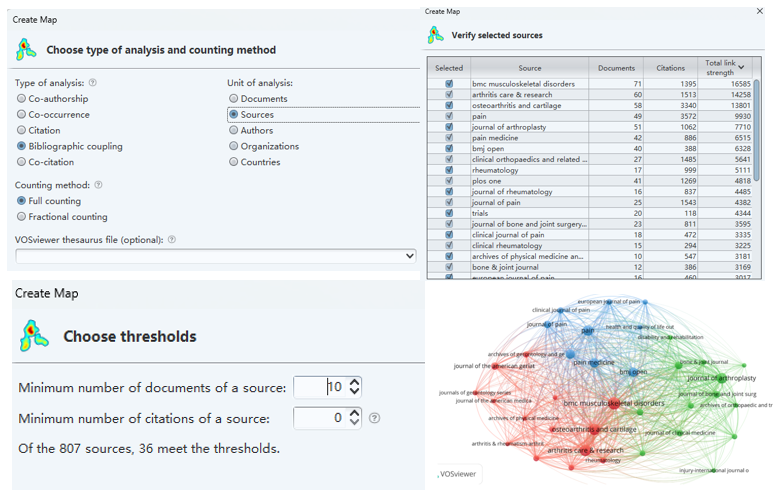


**Figure 4D: Strategic theme map based on R software.**

**
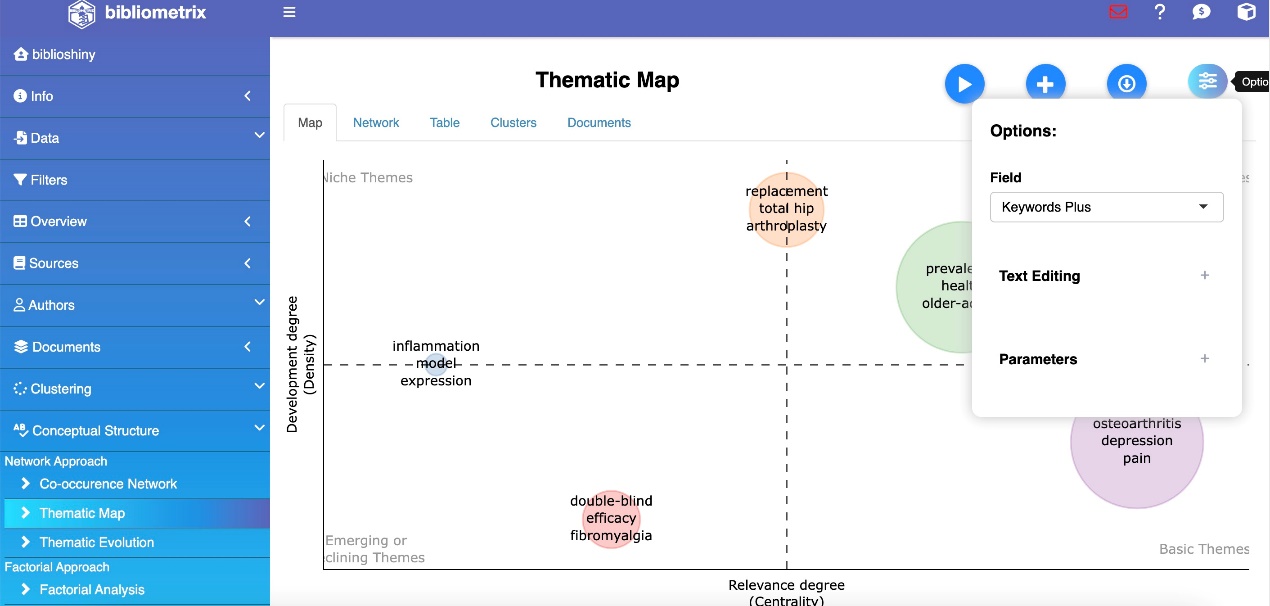
**

**Figure 5A: Mapping of the reference co-citation network in this field.**

**
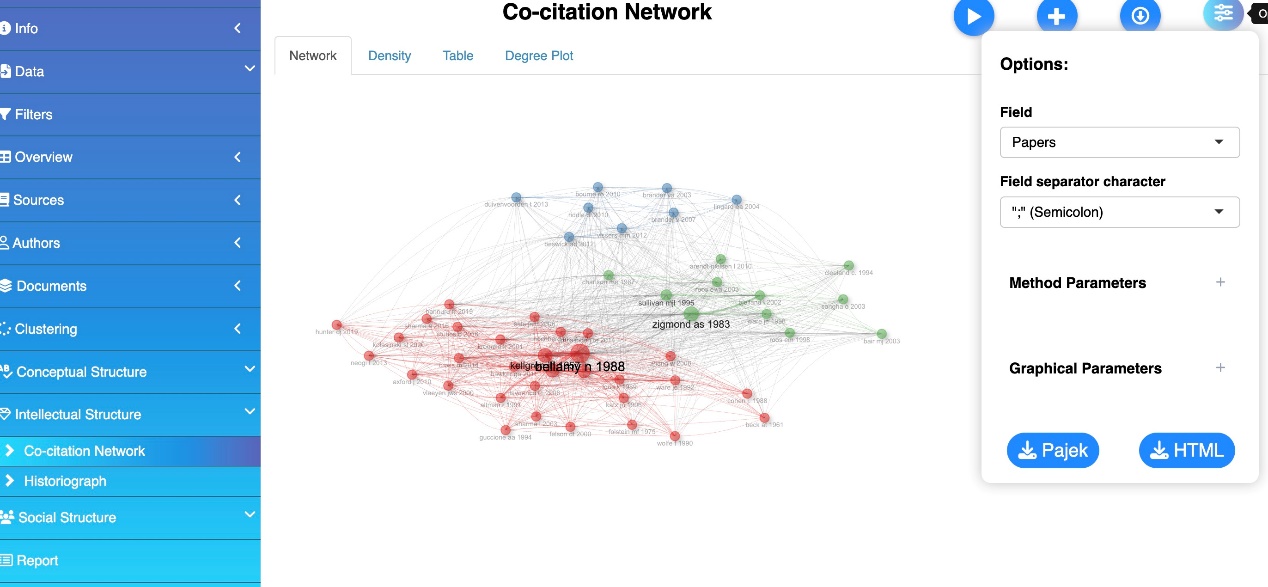
**

**Figure 5B: Mapping of the 147-refencence with citation more than 100 in this field.**


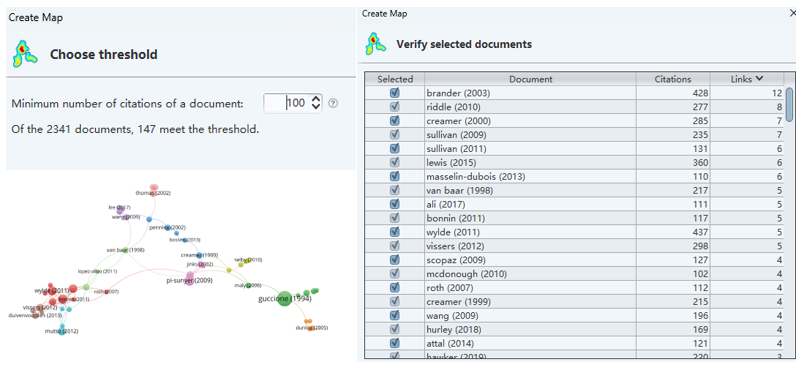


**Figure 5C: Reference growth and decline, the larger the node area, the more important the reference in a given year.**

**
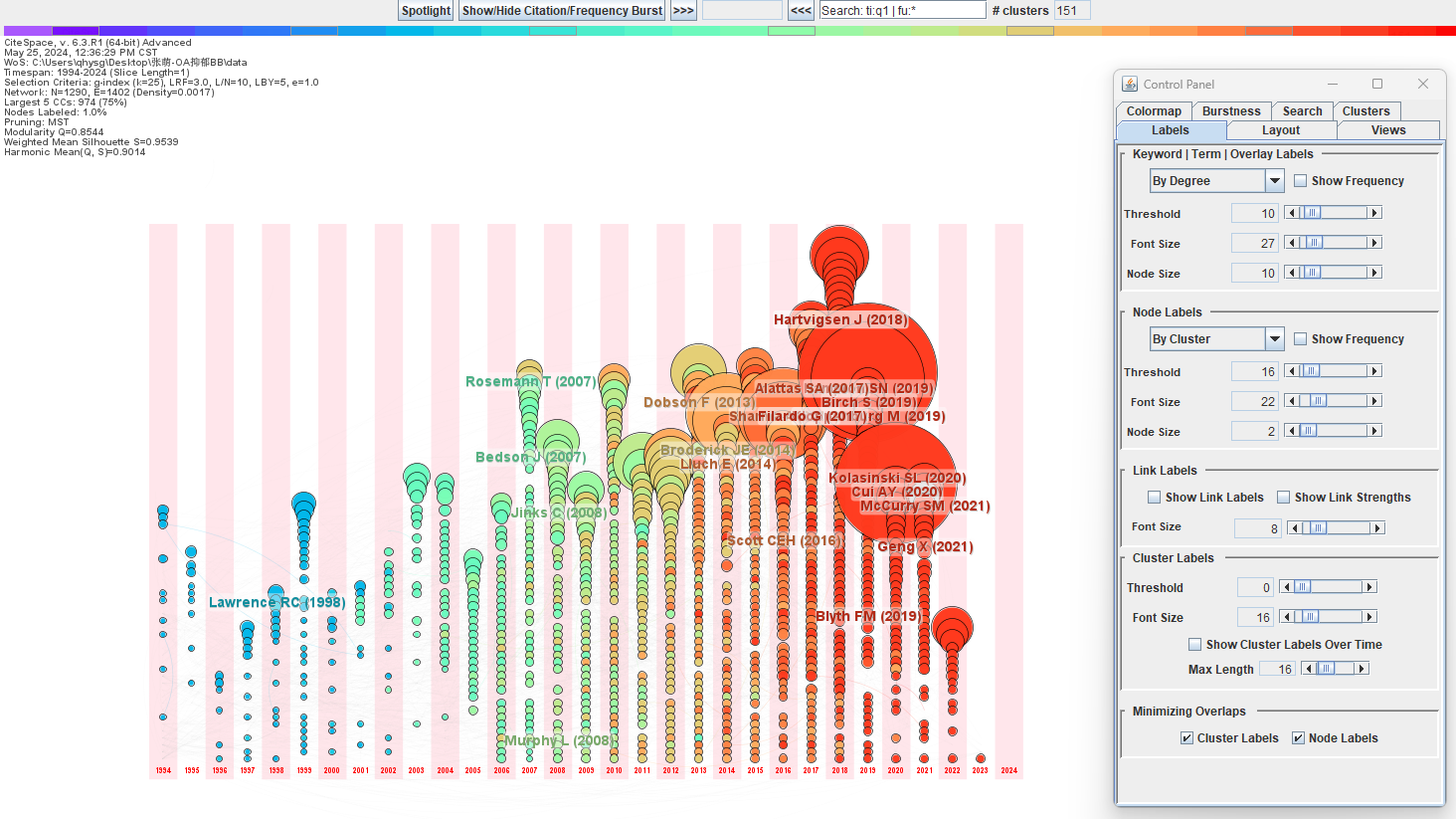
**

**Figure 6A: Co-occurrence analysis of the keyword network based on R software.**

**
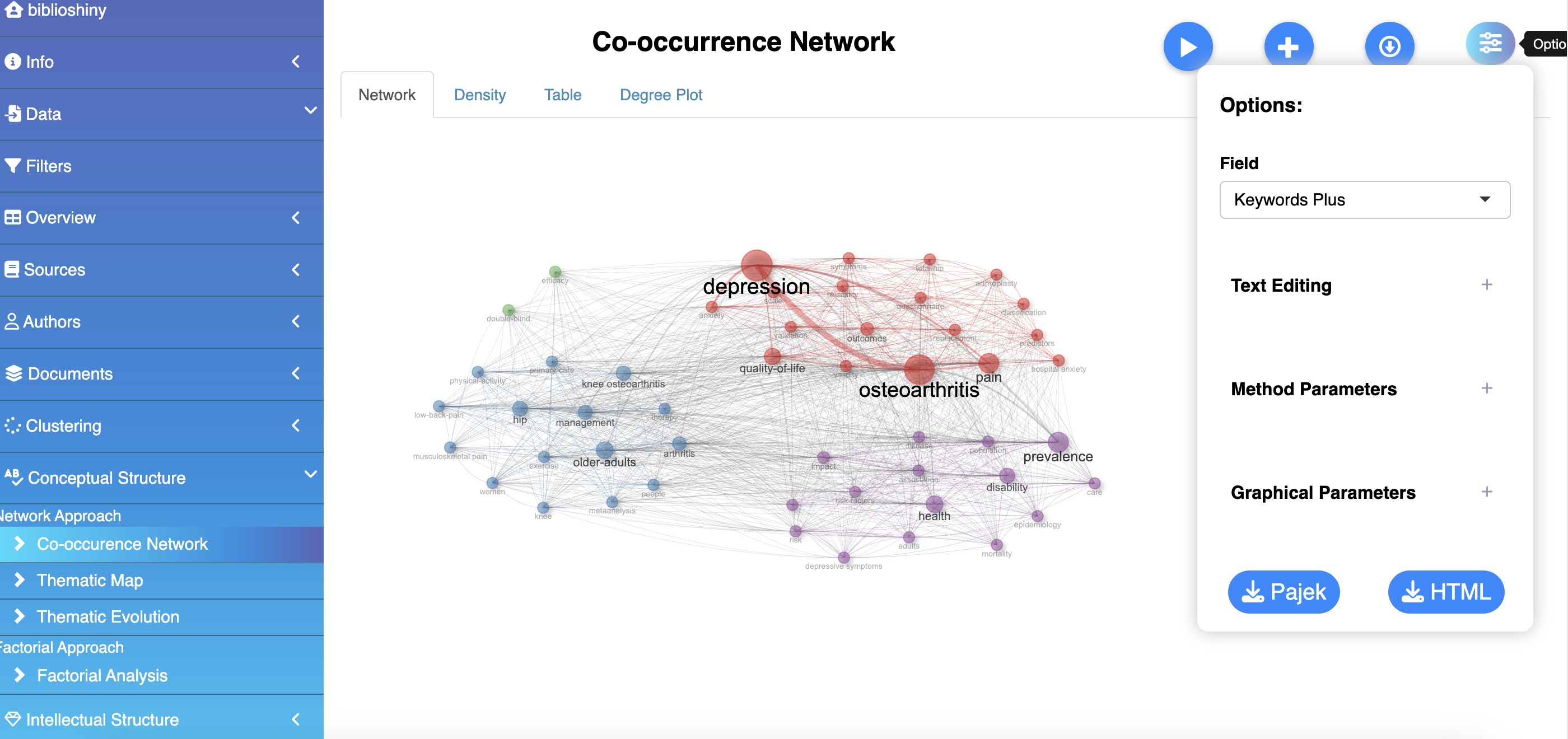
**

**Figure 6B: Distribution of keywords according to average publication year (blue: earlier, yellow: later) by VOSviewer.**


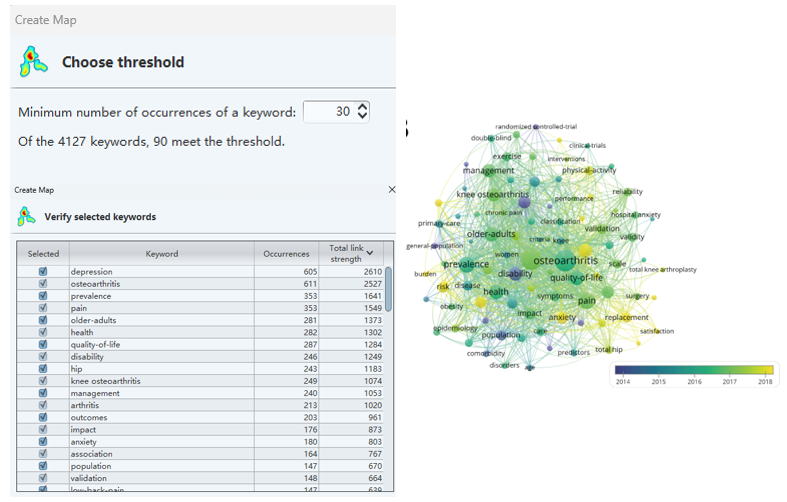


**Figure 6C: Clustering analysis of the keyword network based on CiteSpace.**

**
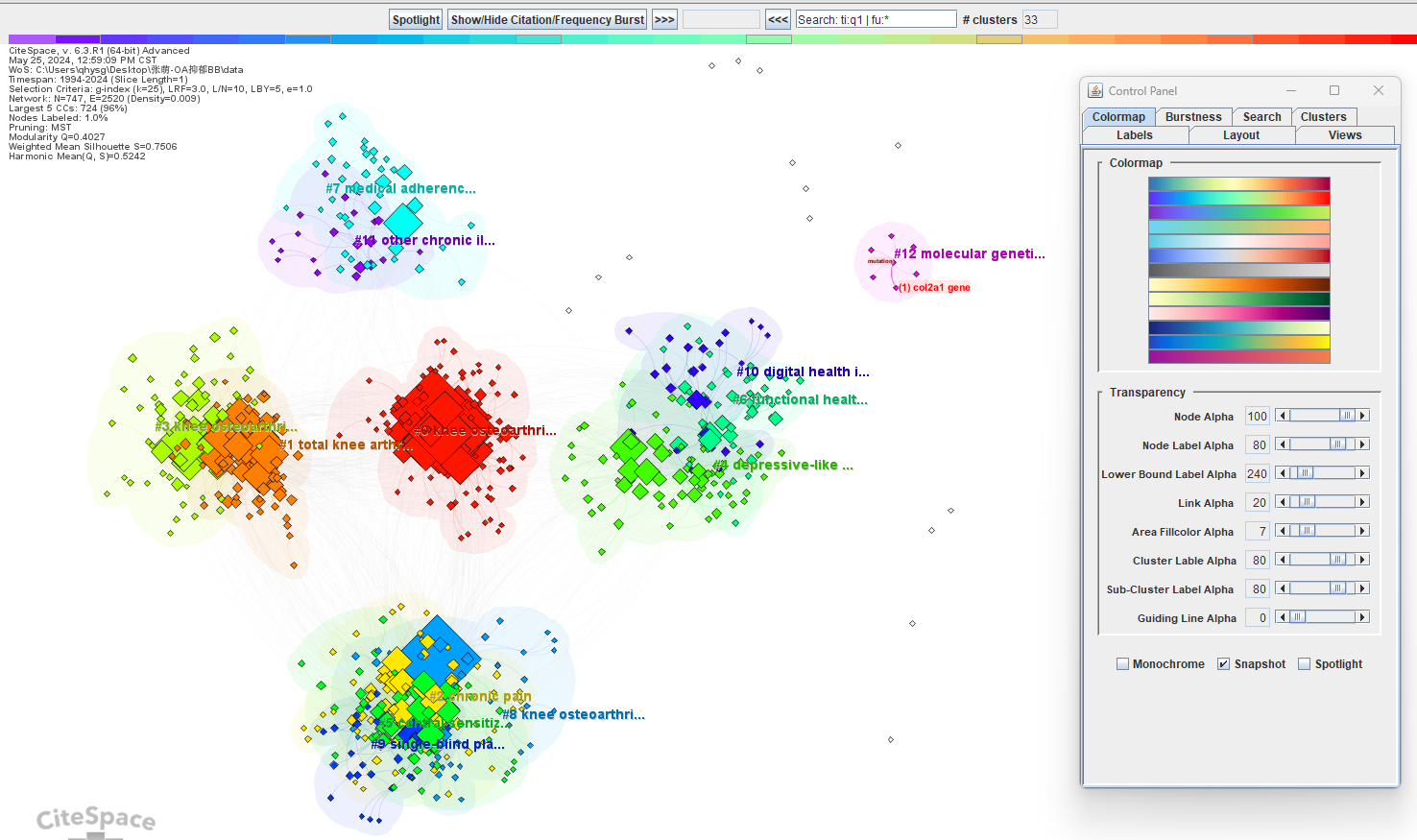
**

**Figure 6D: Keyword timeline visualization from 1994 to 2024 by CiteSpace.**


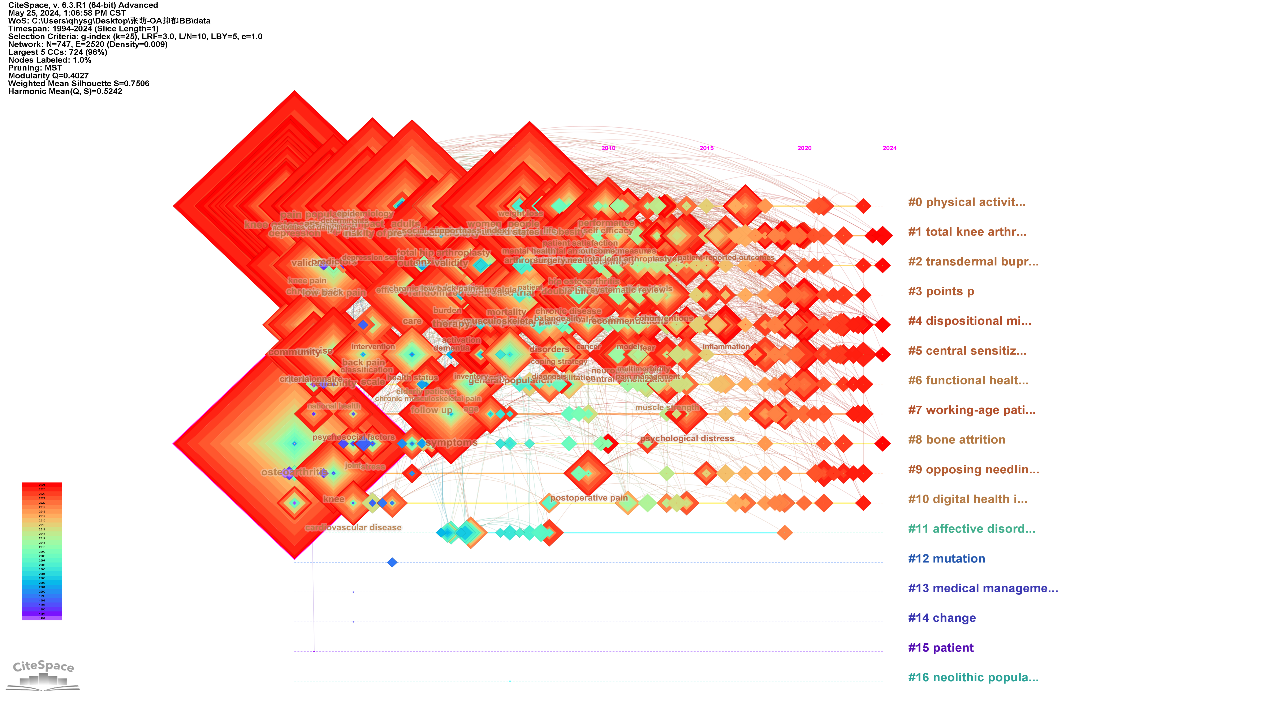

Supplement: Supplementary file 1 [file Table_1.DOCX]
